# TumorDetNet: A unified deep learning model for brain tumor detection and classification

**DOI:** 10.1371/journal.pone.0291200

**Published:** 2023-09-27

**Authors:** Naeem Ullah, Ali Javed, Ali Alhazmi, Syed M. Hasnain, Ali Tahir, Rehan Ashraf

**Affiliations:** 1 Department of Software Engineering, University of Engineering and Technology, Taxila, Pakistan; 2 College of Computer Science and Information Technology, Jazan University, Jazan, Saudi Arabia; 3 Department of Mathematics and Natural Sciences, Prince Mohammad Bin Fahd University, Al Kobar, Saudi Arabia; 4 Department of Computer Science, National Textile University, Faisalabad, Pakistan; University of Manitoba, CANADA

## Abstract

Accurate diagnosis of the brain tumor type at an earlier stage is crucial for the treatment process and helps to save the lives of a large number of people worldwide. Because they are non-invasive and spare patients from having an unpleasant biopsy, magnetic resonance imaging (MRI) scans are frequently employed to identify tumors. The manual identification of tumors is difficult and requires considerable time due to the large number of three-dimensional images that an MRI scan of one patient’s brain produces from various angles. Moreover, the variations in location, size, and shape of the brain tumor also make it challenging to detect and classify different types of tumors. Thus, computer-aided diagnostics (CAD) systems have been proposed for the detection of brain tumors. In this paper, we proposed a novel unified end-to-end deep learning model named TumorDetNet for brain tumor detection and classification. Our TumorDetNet framework employs 48 convolution layers with leaky ReLU (LReLU) and ReLU activation functions to compute the most distinctive deep feature maps. Moreover, average pooling and a dropout layer are also used to learn distinctive patterns and reduce overfitting. Finally, one fully connected and a softmax layer are employed to detect and classify the brain tumor into multiple types. We assessed the performance of our method on six standard Kaggle brain tumor MRI datasets for brain tumor detection and classification into (malignant and benign), and (glioma, pituitary, and meningioma). Our model successfully identified brain tumors with remarkable accuracy of 99.83%, classified benign and malignant brain tumors with an ideal accuracy of 100%, and meningiomas, pituitary, and gliomas tumors with an accuracy of 99.27%. These outcomes demonstrate the potency of the suggested methodology for the reliable identification and categorization of brain tumors.

## 1. Introduction

The human brain serves as the body’s organizational and command center and is a vital component of the nervous system, which is in charge of carrying out daily functions. The brain gathers stimuli or messages from the body’s sense organs, analyses them, and then communicates the result or decision to the muscles. Unrestrained mutation or cell division that results in an anomalous group of brain cells that can disrupt normal brain function and eliminate healthy cells are the causes of brain tumors [1,2]. Fatigue, memory issues, nausea, changes in personality and speech, blurred vision, etc., are among the most typical indications of brain tumors. To identify and classify tumors in brain images, radiologists use a variety of medical imaging modalities [3]. Magnetic resonance imaging (MRI) and computerized tomography (CT) scans are frequently utilized to capture information about several human body parts. As the human brain is a highly sensitive organ, MRI is considered a better image modality for the analysis of brain tumors due to its non-invasive nature. MRI provides detailed information about the structure of the brain after generating various 3D slices from multiple directions and hence, MR is one of the most useful image modalities for automatic medical image analysis [4–7]. The radiologists perform two types of tasks on the brain magnetic resonance imaging (MRI): (i) find whether the brain MR images are tumorous or healthy [8–10], and (ii) classify the tumorous brain MRI scans into different types [11–13]. Usually, the manual detection of brain tumors relies on the expertise of radiologists. Manual detection and classification of brain tumor MR images are challenging because of high variations in shapes and sizes of the same tumor type, the similar appearance of different tumor types [14,15], and the limited availability and expertise of radiologists. Furthermore, manual brain tumor identification and classification is tedious, impractical, time-consuming, and non-reproducible for a huge amount of MRI data. An erroneous analysis of a brain tumor type can cause terrible consequences that can lead to the death of any patient. Moreover, the categorization of brain tumors into their numerous pathological types (multiclass classification) is more problematic than binary classification. A reliable computer-aided diagnostics (CAD) system for brain tumor classification is urgently needed to help radiologists overcome the difficulties of manual brain tumor detection and classification [16,17]. In this study, we concentrate on both the identification (tumor and non-tumor) and classification of brain tumors into benign and malignant, as well as glioma, pituitary, and meningioma tumors.

Generally, brain tumors are of two types: benign (also known as non-cancerous) and malignant (known as cancerous) tumors. Benign tumors originating in the brain are non-progressive and cannot grow in the body. Whereas, malignant is a cancerous tumor that spread quickly to other body parts. Primary malignant tumors, which start in the brain, and secondary malignant tumors, which originate in other body parts and reach the brain, are the two major types of malignant tumors [18]. Moreover, meningioma, pituitary, and glioma are other kinds of brain tumors having a very high occurrence rate [14]. The meningioma tumor is always malignant and usually develops in thin tissues that encircle the brain and spinal cord. Whereas glioma and pituitary tumors can be benign or malignant. Pituitary tumors develop because of the cells’ unbalanced proliferation in the pituitary glands near the brain and the glioma develops in the brain’s glial cells. The categorization of tumors into meningioma, glioma, or pituitary is also very challenging because of the variations in size, form, and intensity [19].

Existing works on brain tumor classification have explored either the traditional machine learning (ML) methods using the handcrafted features or deep learning (DL) methods. Conventional ML approaches utilized in this domain usually comprise of preprocessing, feature selection, feature extraction, and classification. Feature extraction and selection are the most critical phases in any reliable automated brain tumor system as it requires prior understanding of the problem domain [20]. Researchers have used various traditional feature extraction approaches including density histogram, gray level co-occurrence matrix (GLCM), local binary patterns (LBP), bag of word (BoW) model, and Histogram of Oriented Gradients (HOG) using the classifiers like support vector machine (SVM) [21] for brain tumor classification. Traditional ML methods were considered the foundation for the detection and classification tasks. In [22], a computerized system is presented that can distinguish between normal brain tissue and tumor, as well as classify brain tumors into low-grade glioma and high-grade glioma tumors. This system [22] employs k-means as a clustering technique, while the feature extraction and reduction mechanisms rely on Discrete Wavelet Transform (DWT) and Principal Component Analysis (PCA), respectively. Finally, SVM was used for the classification task. However, traditional ML approaches for brain tumor detection are time-consuming as they require handcrafted feature extraction methods. On the other hand, the significance of DL frameworks for achieving better performance over traditional ML models for different image classification and object detection tasks has encouraged scientists to create DL-based CAD methods for brain tumor identification or categorization. The DL models compute the reliable discriminative feature maps due to their automatic feature extraction procedure at the dense level. In the last few years, the research community has employed various DL approaches for brain tumor detection because of their improved performance. In [23], the authors employed a Convolutional Neural Network (CNN) to recognize brain tumors. In [24], the segmentation is accomplished using the UNet framework with ResNet50 as the base model. Furthermore, the multi-class classification of brain tumors is accomplished on the Fighsare dataset using transfer learning (TL) and the NASNet framework.

Existing automated methods for recognising and classifying brain tumours have several drawbacks. Some techniques employ manually defined tumour zones for the identification and categorization of brain tumours, which prohibits them from being fully automated. Due to their ability to automatically extract features, DL techniques are gaining popularity; yet, they need a lot of memory and processing capacity. Additionally, DL algorithms typically produce worse results with tiny datasets, which is particularly typical when dealing with datasets of medical image data. Moreover, the unbalanced figshare dataset was used to assess the current approaches for categorizing brain tumors into pituitary, glioma, and meningioma (pertaining to tumor types). Therefore, it is essential to assess performance employing other common databases and metrics (such as recall, specificity, F-Score, and precision) in addition to accuracy. The variety in size, shape, and location of brain tumors further complicates the identification and classification of various tumor types. To get over these limitations, in the present study we proposed a unique end-2-end TumorDetNet DL-based technique for both brain tumour identification and classification. Our proposed TumorDetNet model is based on an improved Mobilenet model that computes the features map with a Leaky Rectified Linear Unit (LReLU) in the initial two layers which are full convolution (FC) layers with 32 kernels of size 3×3 and in the first residual bottleneck layer followed by 14 residual bottleneck layers with ReLU activation function (AF). Moreover, average pooling (AP) and dropout layers are employed to shrink the size of the network and prevent overfitting. In contrast to several earlier methods [25–27], which needed segmentation of tumors before the feature extraction, our methodology does not require any segmentation of tumor region before features extraction and classification. The major contributions of this work are:

- We introduce a fully automatic TumorDetNet DL framework for the effective detection and classification of brain tumors into multiple types.
- The proposed approach is robust to variations in intensity, location, angle, size, and shape of brain tumors in the MRI.
- Extensive experiments were carried out on six standard datasets including the cross-dataset assessment to demonstrate our model’s superiority and generalizability over current methods for brain tumor detection and classification.

The remaining sections of the article are as follows. The overview of the related work is given in Section 2, and the presented methodology is explained in Section 3. Details regarding the experiments carried out for performance evaluation are provided in Section 4. Finally, Section 5 provides the conclusion of our work.

## 2. Related work

This section presents an investigation of the currently available literature on the identification (detection) and classification of brain tumors. Existing works have suggested either the traditional ML-based approaches [28–30] or DL-based approaches [31–36] for the identification and classification of brain tumors. Furthermore, the goal of TL of DL models is to enhance performance on new tasks by utilizing prior knowledge about tasks that are comparable to the current task. It has significantly improved medical image analysis by overcoming the data scarcity issue, saving time and hardware resources [37–43].

Object detection and classification are considered two of the very significant research areas in medical image analysis. The most extensively utilized ML techniques for brain tumor detection and classification include SVM, Decision Trees (DT), k-Nearest Neighbor (KNN), and Multi-Layer Perceptron (MLP). In [44], the authors used a local histogram-based feature extraction technique for brain tumor identification. The MRI image has been initially split into four quadrants. Then, two sub-bins were created for each histogram bin of each quadrant. Each intensity present in the greatest node is counted in one sub-bin, while the remainder of that quadrant is counted in the other. The extracted new feature and the traditional HOG features have been integrated. Among all of the integrated features, 1024 HoG and 128 Expanded Local Histogram features have been selected using the PCA and employed the Random Forest (RF) and SVM for classification. In [45], the authors used MRI Gaussian and nonlinear scale features because of their robustness to rotation, translation, and noise problems. Each MRI is divided into numerous tiny 8-by-8-pixel MR images for features computation to capture minute details and selected the best features based on variance. Finally, the effectiveness of the suggested hybrid feature vector is evaluated using conventional machine learning classifiers. In [46], the MR scan was first improved using the dynamic fuzzy histogram equalization technique. Then, five distinct local binary variations of each major mode of the brain MRI scan are extracted employing the empirical wavelet transform. The ant-lion feature selection approach was used to choose essential and discriminatory traits while removing the unnecessary ones. An SVM classifier was then employed to determine whether the input MR scan was tumorous or normal.

Effective feature representation is vital to attain better classification performance. Existing research works used a fusion of deep- and handcrafted-features to detect brain tumors [47–49]. In [50], the authors used a fusion of DL and hand-crafted features to detect brain tumors. To get discriminative features from all images, the VGG-19 DL model was used in the transfer learning (TL) setup and these features were then combined with hand-crafted features (such as histogram orientation gradient and LBP) through a serial-based method. Feature fusion was used to combine the hand-crafted and deep features. Finally, logistic regression (LGR), DT, KNN, linear discriminant analysis, and SVM were employed for the classification of healthy and gliomas tumors. This method has a high computational cost because of the use of both handcrafted and deep features with multiple classifiers. In [51], the authors evaluated the performance of the existing DL algorithms such as VGG16, AlexNet, ResNet50, VGG19, and ResNet101 for brain tumor identification using brain MR images. Initially, the pre-trained frameworks (employed in TL setup) are evaluated to find the best suitable DL framework. Then the deep features were extracted employing the best DL model, and finally, both the deep features and hand-crafted features were combined. Moreover, the classification performance of different classifiers such as RF, decision tree, KNN, and SVM was also calculated for comparison. In [52], the authors removed the skull from MRI using a brain surface extraction method. In the next step, the best features among the LBP (hand-crafted features) and capsule network (deep features) were carefully chosen by employing a genetic algorithm. Finally, neural networks, naïve Bayes, and SVM classifiers were used for the tumor grads classification.

There are two main issues with the current brain tumor detection and classification systems based on conventional ML methods. First, it only concentrates on features that are either low-level or high-level. Second, domain-specific expertise and knowledge are needed while using the ML algorithms. Because manual feature extraction is required, brain tumor detection and classification systems may perform less effectively. It is not an easy task and is subject to human error because handcrafted features require substantial domain knowledge (i.e., knowledge about the position and location of the tumor in an MRI). In order to effectively detect and classify brain tumors, it is required to create an automated approach based on both high-level and low-level information without the use of custom features. The automated feature extraction used in DL-based techniques, which is more reliable and effective for detection and classification, addresses these issues. Fully automated end-to-end solutions for classifying brain tumours are offered by DL-based methods. DL frameworks use pooling and convolution (Conv) layers to extract the features from the MR images. In [53], the authors adopted kernel extreme learning machines (KELM) for classification after using CNN to extract the hidden features from MRI images. To evaluate the performance of the ensemble of KELM and CNN framework, a dataset of MRI images (pituitary, meningioma, and glioma tumors) was utilized. In [54], the authors introduced CNN, a computerized technique for the detection of brain tumors using MRI data. The Resnet50 framework was employed in [55] to identify brain tumors. The last five levels of the Resnet50 architecture were removed, and eight new layers were created. Additionally, the effectiveness of the Alexnet, Resnet50, Densenet201, Googlenet, and InceptionV3 frameworks were evaluated in order to identify the system that had the highest level of accuracy for identifying and detecting brain tumors. The authors of [56] trained Faster R-CNN from scratch using MRI brain tumor pictures. Faster R-CNN combines the trained AlexNet DL framework with the region-proposed network (RPN). The AlexNet network served as the foundational model for classifying MRI brain tumours. The RPN received the AlexNet feature map as input. A dataset of 50 brain MRI images was used to evaluate the framework. As we already discussed that DL-based models enhance the performance of brain tumor detection and classification as it automatically extracts both low-level and high-level features for classification. However, the classification of brain tumors through MRI images is very challenging because of the irregularities in the appearance of the tumors (i.e., sizes, intensities, and location). Table 1 presents a high-level overview of the existing state-of-the-art approach.

**Table 1:** Comparison of existing techniques.

Method	Method	Accuracy	Dataset	Limitation
Ismael et al. [29]	Transform domain statistical features, backpropagation NN	91.9%	CE-MRI	Low accuracy
Badza et al. [31]	CNN	96.56%	CE-MRI	Low accuracy
Ahamed et al. [32]	CNN with and without TL	99.75%	Br35h: Brain tumor detection	Testing on an imbalance dataset
Irmak et al. [33]	CNN	99.33% 92.66% 98.14%	RIDER, REMBRANDT, TCGA-LGG	Low generalization ability of models
Afshar et al. [34]	Capsule network	86.56%	CE-MRI	Low accuracy Low performance on the entire image
Qureshi et al. [35]	Deep features + GLCM-SVM	99.23%	CE-MRI	Overfitting on larger size (256 × 256) images
Hassan et al. [48]	Deep features + GLCM-SVM	99.0%	BRATS 2013 MRI	Features of deeper TL models degrade the performance
Oksuz et al. [49]	Deep features (pre-trained AlexNet, ResNet-18, GoogLeNet, ShuffleNet)-SVM+KNN.	97.25%	CE-MRI	The method is tested on an imbalance dataset
Saba et al. [50]	VGG-19+LBP+HoG-logistic regression, DT, K-NN, LDA, SVM	98.78%	BRATS 2016	High computational cost
Sharif et al. [51]	LBP + deep features-ANN	98.34%	RIDER and BRATS 2018	Low classification accuracy in case of deep features
Cinar et al. [55]	Resnet50	97.01%	Brain Tumor Detection	Low classification accuracy
Ezhilarasi et al. [56]	AlexNet+Faster R-CNN	99.0%	Brain tumor MRI (Radiology Assistant dataset)	The model trained on a small dataset
Kibriya et al. [47]	CNN	97.2%	CE-MRI	Low classification accuracy

Since most methods employ limited or unbalanced datasets, the existing research on brain tumor detection and classification is unable to achieve superior detection and classification performance. Brain tumors come in various sizes, forms, and locations, making it more difficult to identify and classify different types of tumors, and ultimately results in performance degradation. Therefore, more reliable methods and diverse, balanced, and large-scale datasets are urgently needed to solve brain tumor detection and classification constraints.

## 3. Methodology

**Fig 1:**
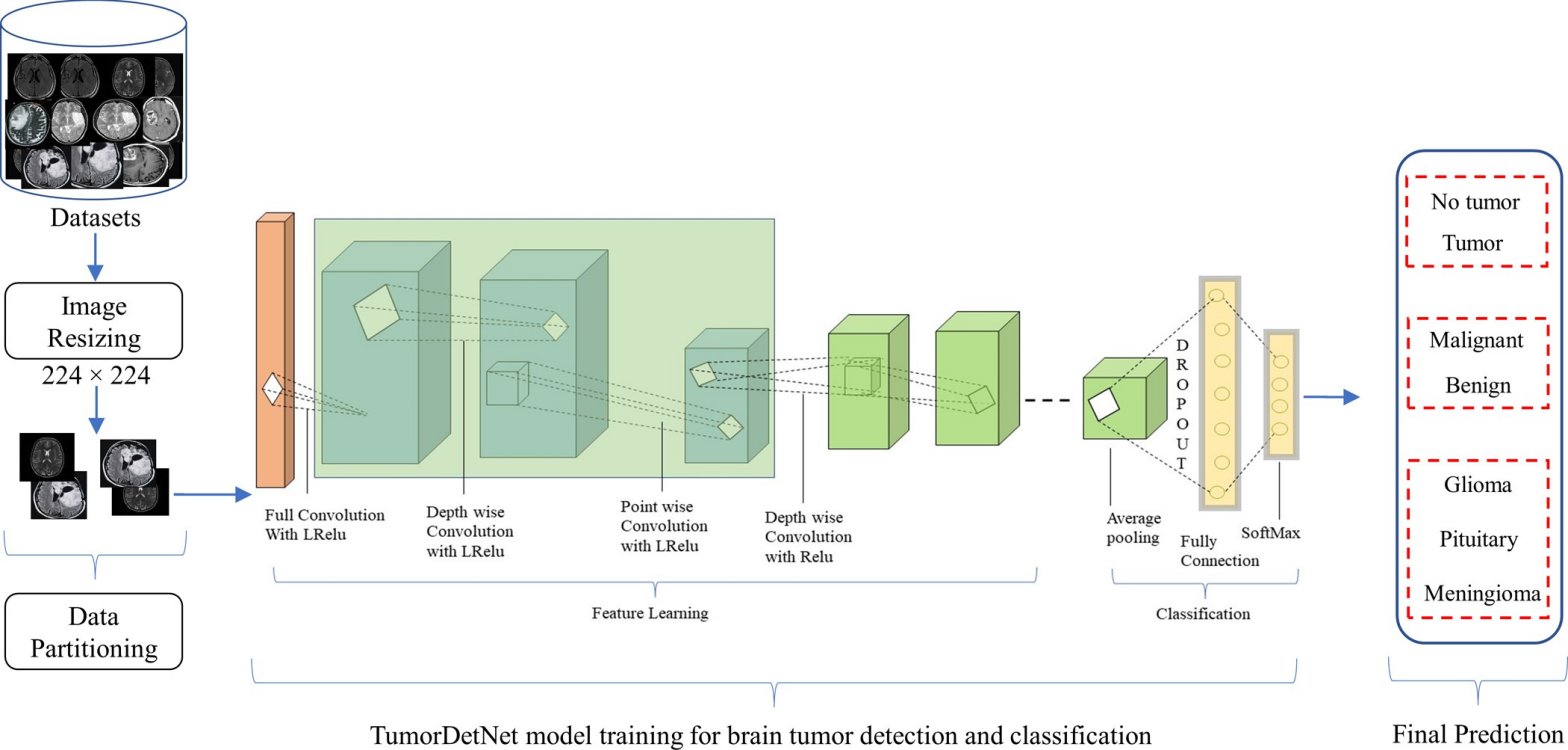
Process flow of the proposed system.

This work presents a novel TumorDetNet model to automatically identify and classify brain tumor MRI scans. Our work includes two stages: a) brain tumor identification, and b) tumor classification. We perform both the two-class (benign and malignant) and three-class (meningioma, pituitary, and glioma) classification in this work. Inspired by the success and feature extraction capabilities of Mobilenet in the area of object detection and classification, we proposed an improved MobileNetv2 model for the discovery and extraction of more reliable and robust deep features. The AP, FC, and softmax layers are employed for the classification of brain tumor MRI data. The overall process is shown in Fig 1.

### 3.1 Motivation of the proposed TumorDetNet model

Googlenet [57] and Mobilenet [58] are the most extensively employed CNN architectures for image classification applications. Inspired by the classification ability of Googlenet and feature extraction capabilities of Mobilenet architecture, we proposed a novel TumorDetNet model for brain tumor detection and classification in this study. Developing an end-2-end DL framework without prior tumor segmentation and capable of reliable detection and classification of brain tumors was the core motivation behind this study. Our proposed TumorDetNet model presents an improved MobileNetv2 with both the LReLU and ReLU AFs [59] for effective features map generation. LReLU activation is used to solve the issue of the dying ReLU problem. Next, AP and dropout layers are employed to further refine the features and reduce overfitting. Finally, an FC and a softmax layer are used for the classification of MRI data. The details of MobileNetv2 and GoogleNet base models are briefly described in this section.

MobileNetv2 [58,60–62] is a lightweight DL-based model as compared to other frameworks, which makes this DL framework more appropriate for real-time tasks. The performance of the Mobilenetv2 model increases because of depthwise separable convolutions (a form of factorized Conv). Depthwise separable convolutions have a lesser computational cost compared to traditional convolutions. Depthwise separable convolutions split the standard Conv into a depthwise and pointwise Conv with a 1 × 1 Conv. Depthwise Conv means that each of the filters performs a single Conv on each color channel rather than combining all the three-color channels, thus filters the input channels. By using bottleneck layers to reduce the input size, the computation time is greatly decreased. In the MobileNet architecture, the layers are connected by the ReLU AF [58], which enables the non-linear outputs from one layer to be flattened and provided as input to the next layer. Two hyperparameters are presented in MobileNet for shrinking and factorizing, i.e., named width multiplier (*α*) and resolution multiplier (*ρ*). Both the values of *α* and *ρ* are in the range (0, 1). The value of *α* is used to reduce the number of filters, while the value of *ρ* is used to reduce the image resolution. Thus, we can reduce the computational cost by altering the values of *α* and *ρ*.

GoogLeNet [57] was developed in 2014. GoogleNet was trained on the ILSVRC dataset and was the first winner of ILSVRC 2014. There are 22 layers in the architecture. The architecture is made up of nine inception modules, two Conv layers (also one more Conv layer for dimension-reduction), four max-pooling layers, two normalization layers, an AP layer, an FC layer, and lastly a linear layer with softmax activation at the output. Approximately 6.8 million parameters make up the model. Each inception module also includes a max-pooling layer in addition to six Conv layers, of which 4 are used for dimension reduction. The fully FC layers employ dropout regularization to prevent overfitting and increase the framework’s effectiveness.

Furthermore, our model’s number of layers is based on the well-known idea of depth scaling, which is utilized to increase accuracy. The logical assumption is that deeper CNNs extract richer, more complex features and generalize effectively to new data, leading to better model performance. Though there is no guarantee of greater accuracy in all scenarios, the computational cost rises as the network’s depth raises as well. Deeper networks are more challenging to train due to the vanishing gradient issue. Even though there are several solutions to the training problem, such as skip connections and batch normalization, the accuracy gain of deep networks reduces over time. For instance, ResNet-1000 has equal accuracy to ResNet-101 while having many more layers. We used batch normalization in the TumorDetNet architecture to solve the vanishing gradient issue.

### 3.2 Proposed TumorDetNet model

**Fig 2:**
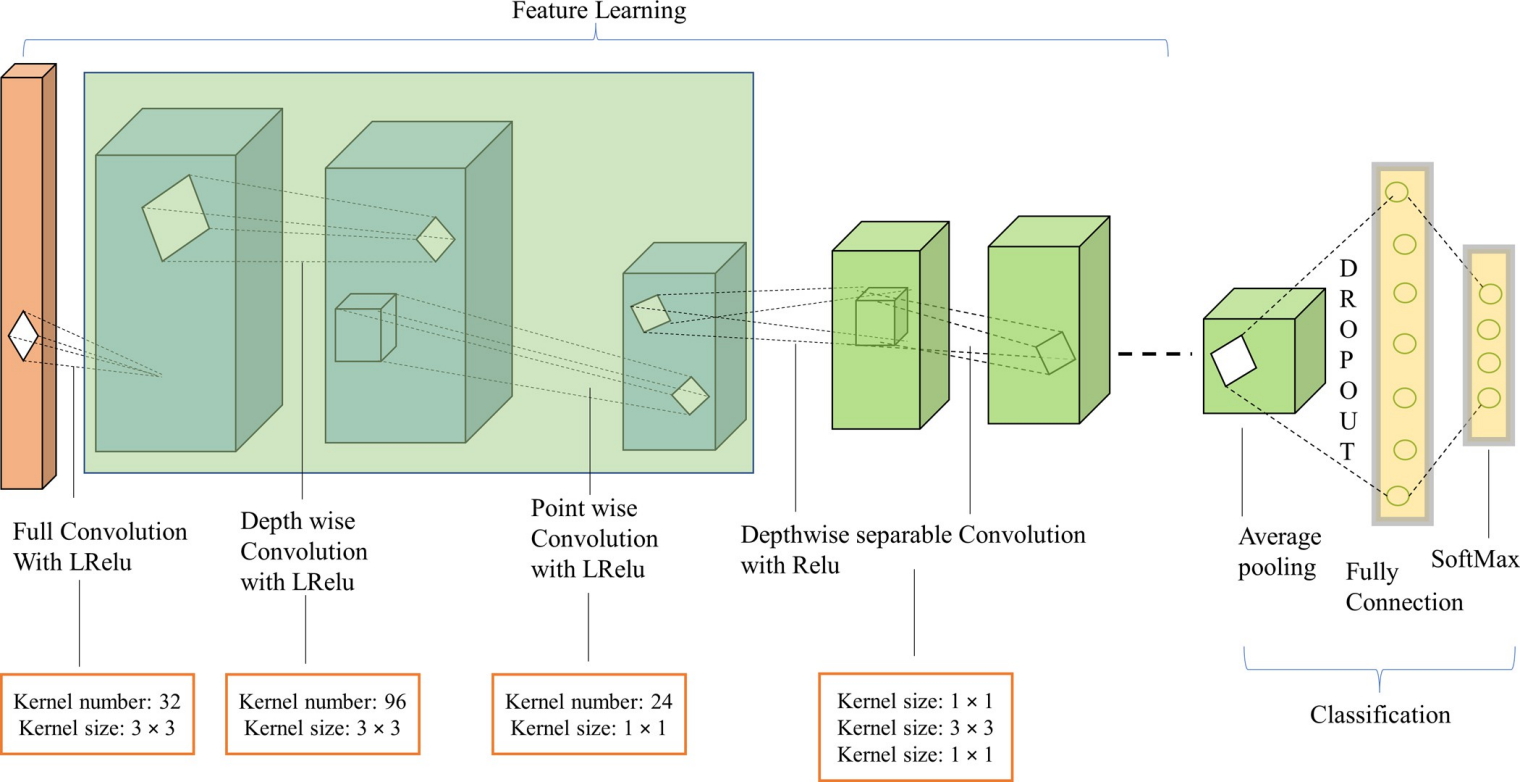
Architecture of the proposed TumorDetNet model.

In this study, we proposed a novel end-2-end TumorDetNet DL framework for brain tumor identification and classification. The proposed model uses 129 layers (i.e., Conv layers and group convolutions, normalizations, and AFs) for feature extractions and 5 layers (including global AP, dropout, FC, Softmax, and classification) for brain tumor classification. Our framework is deeper than typical CNN with 49 learnable layers: 48 Conv layers and one FC layer as revealed in Table 2. The initial layer of our model is the image input layer which receives 224×224 input images for processing so we down-sampled the input frames to 224 × 224 to decrease the computational complexity of our model. The model has a total of 134 layers including 48 batch normalization (BN), 4 LReLu, 28 ReLU, one global AP (to reduce model parameters), dropout, FC, Softmax, and a classification layer each. A total of four LReLU and twenty-eight ReLU layers are used. To reduce overfitting, a dropout of 0.4% is applied to the FC layer. We used the stride of 2 × 2 in five convolutional layers to reduce spatial resolution leading to computational benefits. The proposed architecture contains the first fully Conv layer with 32 kernels followed by 15 residual bottleneck layers. The initial fully convolutional and the first residual bottleneck layer are followed by the LReLU layers whereas the rest 14 residual bottleneck layers are followed by the ReLU AF. The residual bottleneck layers consist of depthwise convolution (with a kernel of size 3 × 3) and a pointwise Conv (with a filter of size 1 × 1). The small convolutional filters are utilized to extract the most important features from the MRI data. In the residual bottleneck layers of our proposed model, the first Conv (with a 1 × 1 filter) is followed by the BN layer (with an epsilon of 0.001) and AF (LReLU in the case of first residual bottleneck layer and ReLU in case of the rest 14 residual bottleneck layers). Similarly, the BN and AF come after the second Conv layer (which has a 3 × 3 kernel). The BN layer immediately follows the third Conv layer (with a 1 × 1 kernel), though. After feeding an FC layer with the features recovered using the residual bottleneck layers, Soft-max activation is used to calculate the classification probability. Due to the usage of filters with a size of 1, our model successfully recovers the most discriminative and fine-level characteristics. Additionally, our model’s depth-wise separable convolutions increase efficiency by reducing the number of parameters and calculations required for Conv operations. The dropout layer also stops the model from overfitting. The proposed model’s structure is shown in Fig 2.

**Table 2:** Architectural details of the proposed DL framework.

S No	Operator	Layers	Filter	No of filters	Padding	Stride	n
1	Conv	Conv (BN, LRelu)	3 × 3	32	same	2 × 2	2
2	Conv	Conv (BN)	1 × 1	16	Same	-	1
3	Bottleneck	Conv (BN, LRelu)	1 × 1	96	same	-	1
		Conv (BN, LRelu)	3 × 3	96		2 × 2	
		Conv (BN)	1 × 1	24		-	
4	Bottleneck	Conv (BN, Relu)	1 × 1	144	same	-	2
		Conv (BN, Relu)	3 × 3	144		2 × 2	
		Conv (BN)	1 × 1	24		-	
5	Bottleneck	Conv (BN, Relu)	1 × 1	192	same	-	2
		Conv (BN, Relu)	3 × 3	192		-	
		Conv (BN)	1 × 1	32		-	
6	Bottleneck	Conv (BN, Relu)	1 × 1	192	same	-	1
		Conv (BN, Relu)	3 × 3	192		2 × 2	
		Conv (BN)	1 × 1	64		-	
7	Bottleneck	Conv (BN, Relu)	1 × 1	383	same	-	3
		Conv (BN, Relu)	3 × 3	383		-	
		Conv (BN)	1 × 1	64		-	
8	Bottleneck	Conv (BN, Relu)	1 × 1	383	Same	-	1
		Conv (BN, Relu)	3 × 3	383		-	
		Conv (BN)	1 × 1	96		-	
9	Bottleneck	Conv (BN, Relu)	1 × 1	576	same	-	2
		Conv (BN, Relu)	3 × 3	576		-	
		Conv (BN)	1 × 1	96		-	
10	Bottleneck	Conv (BN, Relu)	1 × 1	576	same	-	1
		Conv (BN, Relu)	3 × 3	576		2 × 2	
		Conv (BN)	1 × 1	160		-	
11	Bottleneck	Conv (BN, Relu)	1 × 1	690	same	-	2
		Conv (BN, Relu)	3 × 3	690		-	
		Conv (BN)	1 × 1	160		-	
12	AP		7 × 7				
13	Dropout (0.4)						
14	FC + Softmax + classification						

Activation functions have a central role in deciding the firing of a neuron in network learning. Rectified linear unit (ReLU) [59] is a non-linear AF, which is computationally efficient (because it doesn’t need to perform complex exponential operations; it simply needs to select the maximum (0, x) value) and is very effective, especially in deep neural networks. The ReLU AF outputs zero for negative inputs (x<0), whereas, outputs the non-negative input directly. Additionally, a 0 value on the negative axis means that the model will operate more quickly. However, the ReLU AF does not activate the neuron in response to negative inputs. In this case, the optimization algorithm cannot help the network learn (dying ReLU problem). Because a major component of the model becomes gradually inactive, the dying ReLU problem is undesirable. In order to address the dying ReLU issue, we used the LReLU [63] AF in the initial full Conv layer. LReLU assigns a small positive value, for example, 0.1 instead of 0, to maintain all the neurons active for the majority of the training samples. The LReLU AF helps in increasing the ReLU function’s coverage area. LReLU ensures that all neurons of the model must contribute to the active performance of the network. Since the performance of DL frameworks is greatly influenced by the activation functions, therefore, we used both the LReLU and ReLU within the feature map of our proposed model to resolve the dying ReLU issue and to make our model computationally efficient, respectively.

### 3.3 Hyper-parameters

The selection of hyper-parameters is a key factor in the better optimization of any DL framework [64]. Hyper-parameters include epoch size, AF, size of the learning rate, kernel, minibatch size, etc. The choice of these hyper-parameters is important, as it influences the model’s functionality [64]. Before beginning the training process, hyper-parameters must be chosen because they are different from the model components. Given the wide range of options for hyper-parameters, we assessed the performance of the suggested framework using several hyper-parameter values to find the optimal values. After performing experiments on various hyperparameters, we selected those parameter settings where we attained the best results. Details of the chosen hyper-parameters are listed in Table 3 lists the details.

**Table 3:** Parameters of the proposed architecture.

Parameter	Value
Optimization algorithm	SGDM
learning rate	0.01
Maximum Epochs	22
Shuffle	Every epoch
Validation frequency	30
Iterations per epoch	42
Verbose	False
AF	Leaky ReLU + ReLU
Dropout	0.4
Train Size	0.8
Test Size	0.2

## 4. Experiments and results

This section presents an in-depth explanation on the results of several experiments designed to assess the classification performance of the proposed method. Moreover, information about all the six datasets (Table 4) used for brain tumor identification and classification is also provided in this section.

**Table 4:** Datasets details.

Purpose	Dataset	Tumor type			
Detection	TD-MRI	**Tumor**		**No tumor**	
		1500		1500	
	BMI-BTD	155		98	
2-Class classification	TCD	**Benign**		**Malignant**	
		350		350	
	BTTypes	1200		1200	
		**Meningioma**	**Pituitary**		**Glioma**
3-Class classification	BTC	937	898		926
	CE-MRI	708	930		1426

### 4.1 Datasets

We utilized six datasets in this work to assess our model’s performance. Two datasets are utilized for brain tumor detection, two for brain tumor classification into benign and malignant, and two for the classification into glioma, pituitary, and meningioma tumors. All the datasets utilized in this work contain grayscale images of different resolutions.

#### 4.1.1 Brain tumor datasets

**Fig 3:**
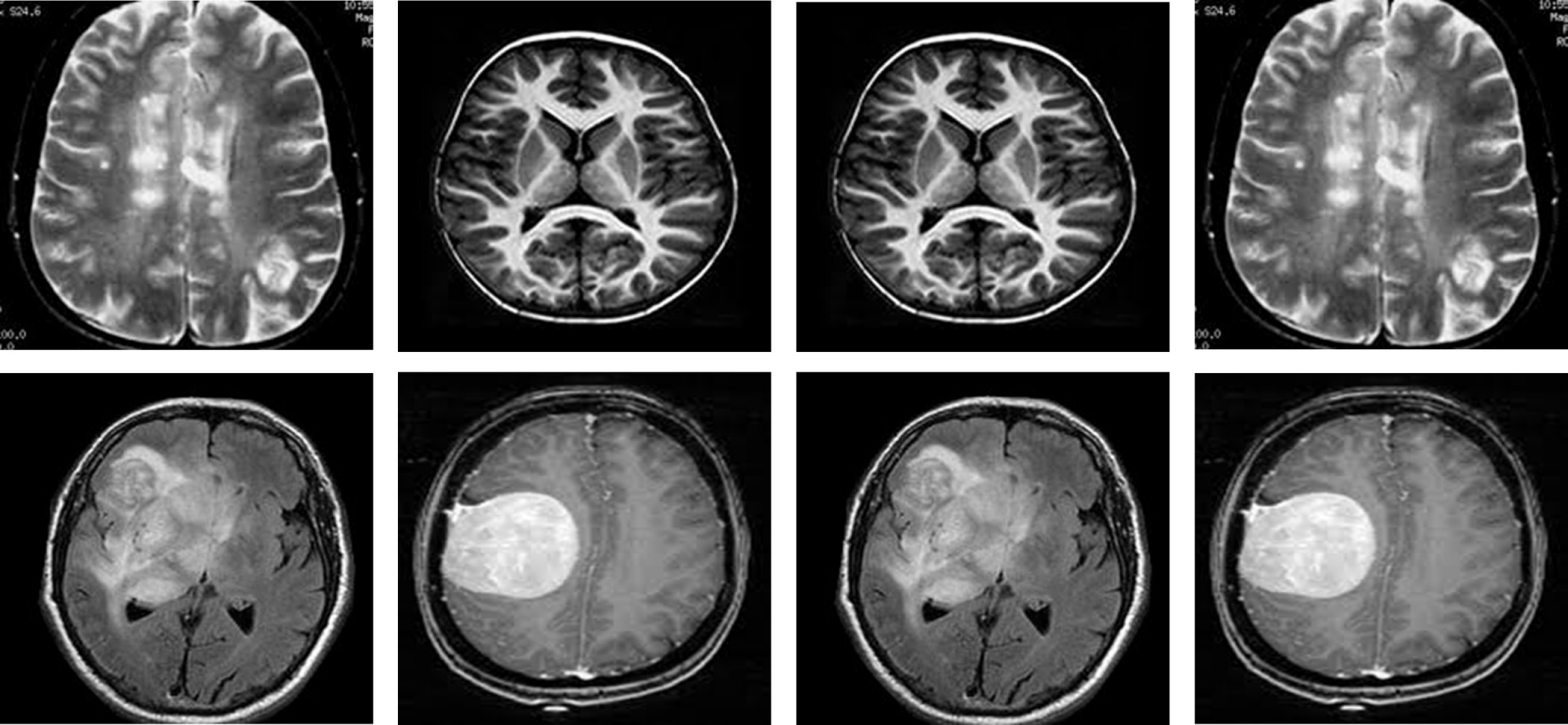
Samples of BTD-MRI dataset, upper row: Non-tumor, lower row: Tumorous.

**Fig 4:**
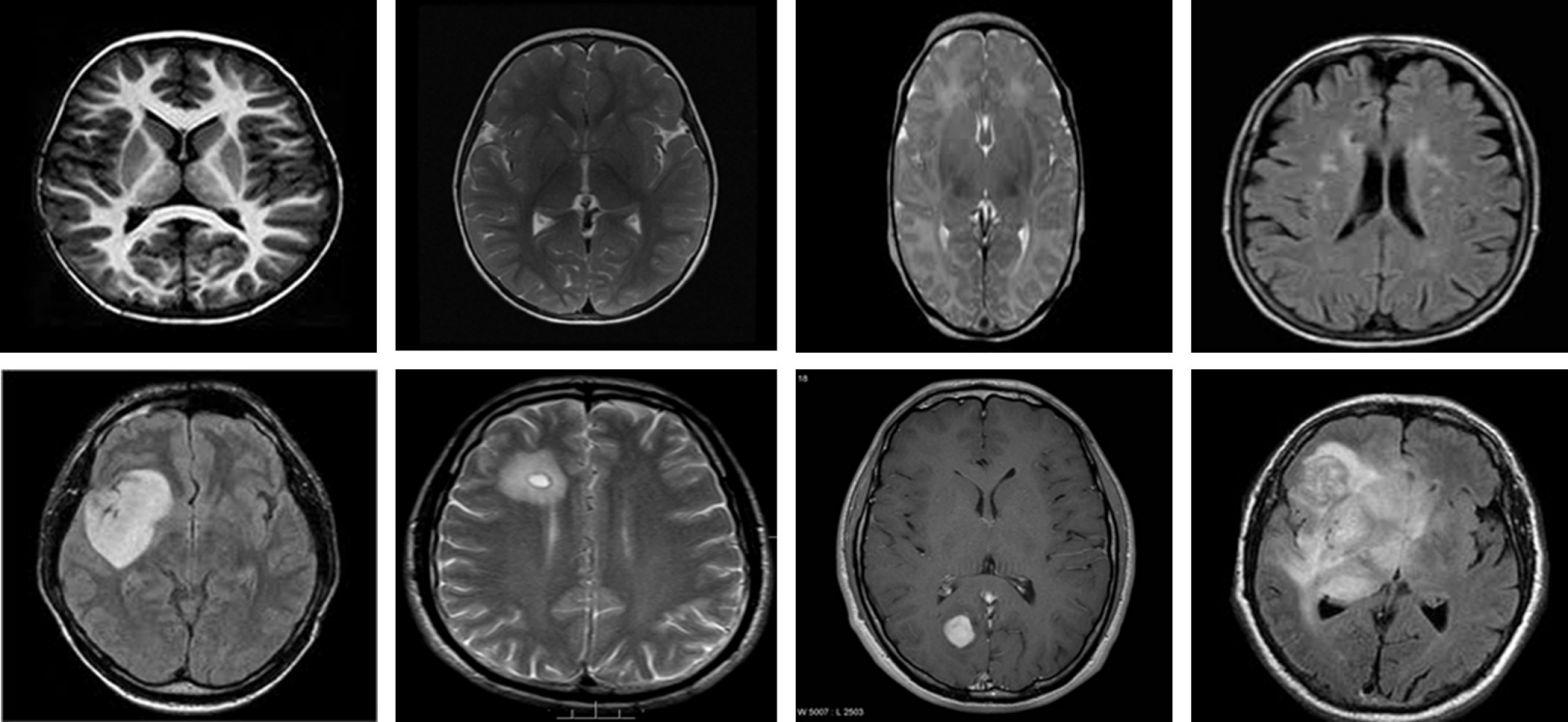
Samples of BMI-BTD dataset, upper row: Non-tumor, lower row: Tumorous.

We utilised the Tumor_Detection_MRI (TD-MRI) database, which is publicly available on Kaggle [65], to identify brain tumours. There are two collections in the database. The second collection has 1500 MRI scans of tumours, whereas the first collection contains 1500 MRI scans of tumors. Fig 3 displays a small number of samples from this collection. The second dataset used for brain tumor identification is Brain MRI scans for Brain Tumour identification (BMI-BTD), which is another publicly downloadable standard Kaggle database [66]. The dataset consists of two collections, the first of which contains 98 MRI pictures free of tumours and the second of which has 155 such images. Fig 4 displays a small number of samples from this collection.

#### 4.1.2 2-class classification datasets

**Fig 5:**
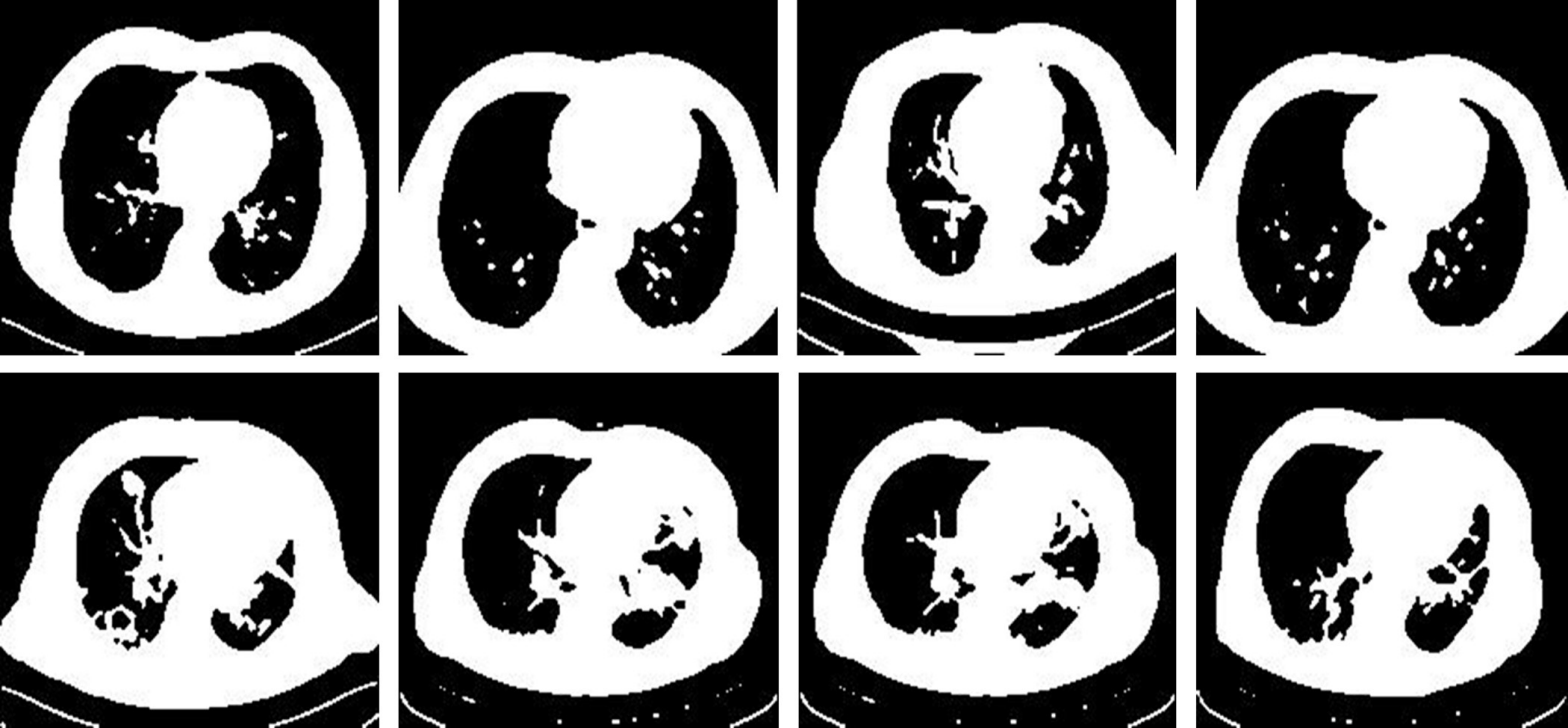
Samples of TCD dataset, upper row: Benign, lower row: Malignant.

**Fig 6:**
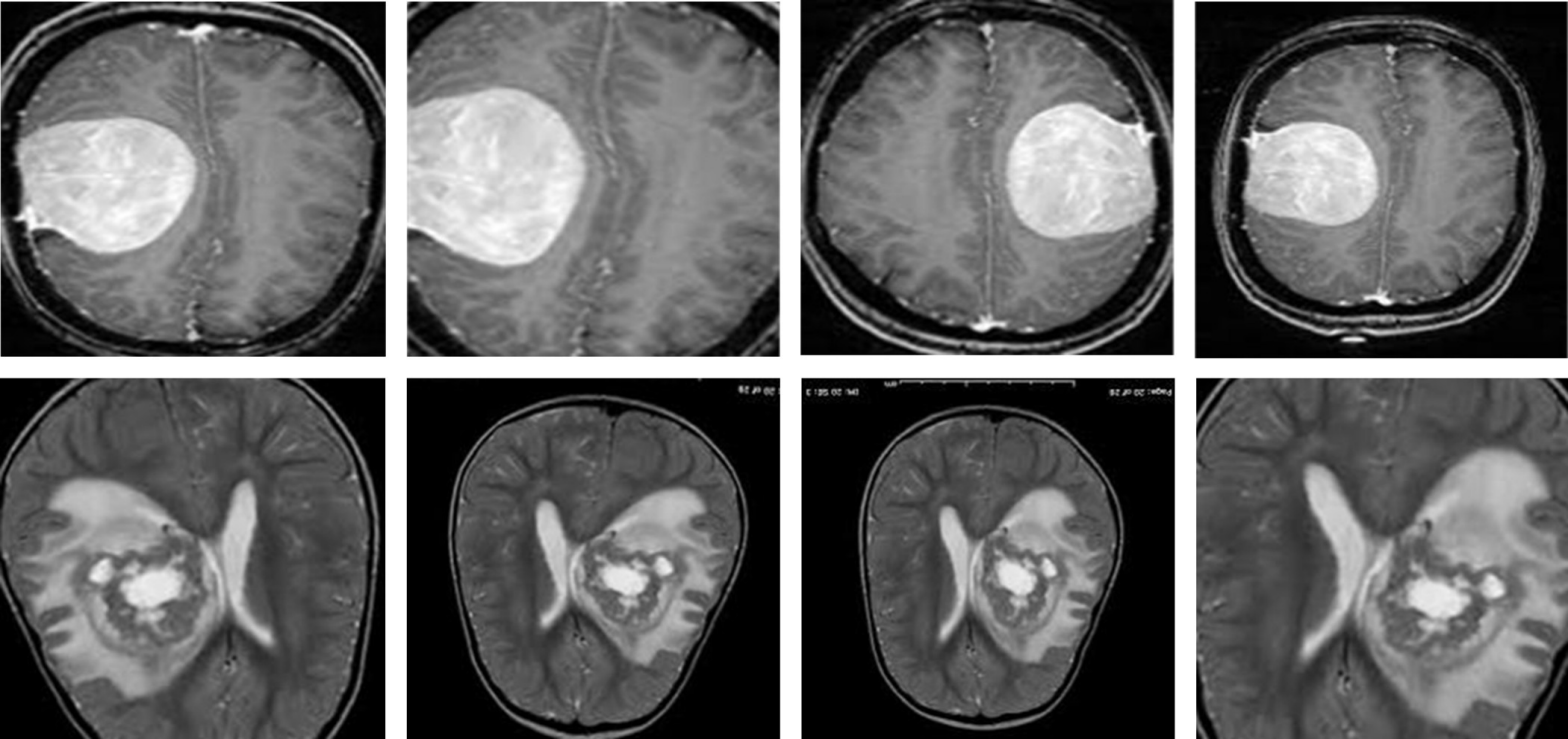
Samples of BTTypes dataset, upper row: Benign, lower row: Malignant images.

The dataset for the classification of brain tumors into benign and malignant used in this work is taken from the Tumor Classification Data (TCD) freely available on Kaggle [67]. This database has MRI scans of the malignant and benign tumors along with healthy brain images. The database comprises two groups, i.e., train and test, each having three sub-collections of benign, malignant, and normal. We used only benign and malignant collections, each having 350 images. We used all the 700 images (350 benign and 350 malignant) for the experimentation. Few samples of the database are shown in Fig 5. The second database (BTTypes) is utilized for brain tumor classification and is freely available at Kaggle [68]. The dataset comprises two collections, i.e., benign and malignant, each having 1200 images. We used all the 2400 images (1200 benign and 1200 malignant) for the experimentation. Samples of the database are shown in Fig 6.

#### 4.1.3 3-class classification datasets

**Fig 7:**
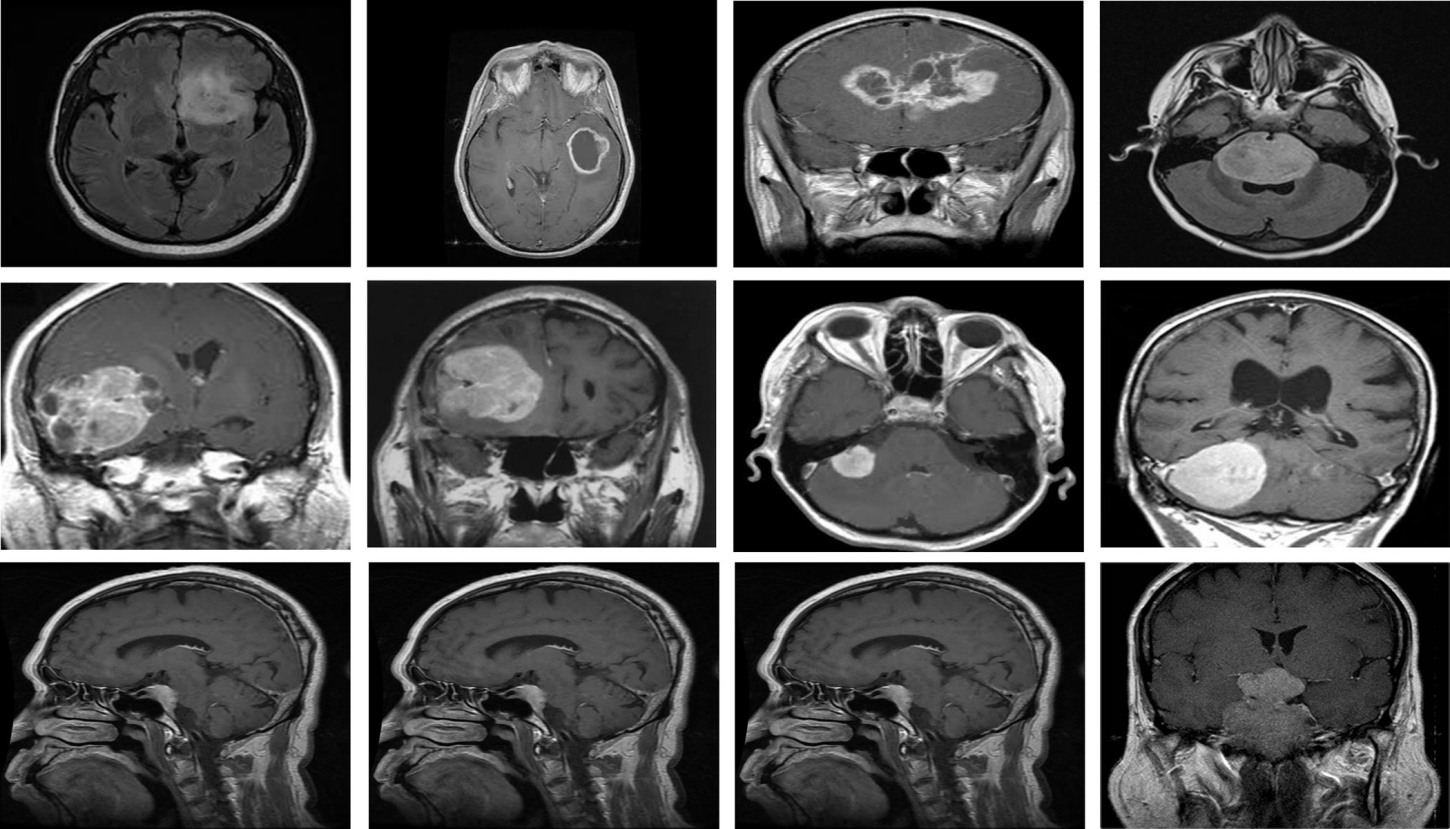
Samples of BTC dataset, upper row: Glioma tumor, middle row: Meningioma tumor, lower row: Pituitary tumor.

We used the Brain tumor classification dataset (BTC), accessible at Kaggle [69], to categorize brain tumors into meningioma, pituitary, and glioma. The dataset consists of training and testing collections of brain tumor MRI images. There are four different categories of brain tumor MRI scans in each folder, including no tumors, gliomas, pituitary, and meningiomas. However, we only made use of the MRI scans of the pituitary, glioma, and meningioma tumors. The training collection of the dataset’s most recent version includes 822 MRI scans of meningiomas, 827 MRI scans of pituitary, and 826 MRI scans of glioma tumors. In contrast, the testing collection includes 100 images of glioma, 74 images of the pituitary, and 115 images of meningiomas tumors. We merged images of both collections and then used the images for training and testing. Some images of this dataset are shown in Fig 7.

**Fig 8:**
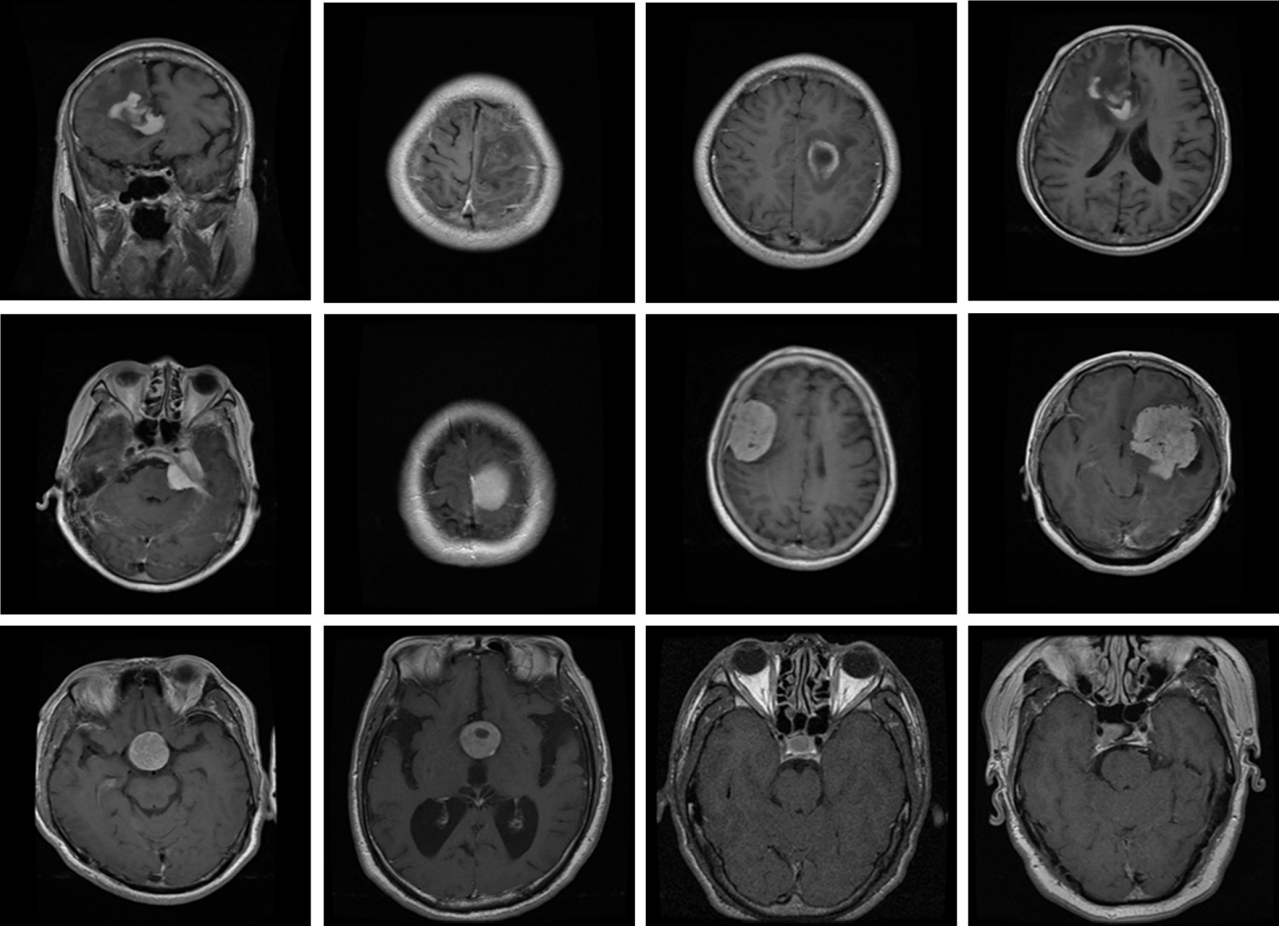
Images from the CE-MRI dataset, upper row: Glioma, middle row: Meningioma, lower row: Pituitary.

The second dataset used for the classification of brain tumors into meningioma, pituitary, and glioma is the publicly available CE-MRI Figshare dataset [70] which comprises a total of 3064 2D MRI scans with T1-weighted contrast-enhanced modality attained from 233 affected individuals. Three classes i.e., pituitary, glioma, and meningioma represent the categories of brain tumors, and the T1 modality emphasizes specific characteristics of each. The most recent version of this dataset includes 930 images of pituitary, 1426 images of glioma, and 708 images of meningioma. The collection includes MR images of 512-by-512-pixel size. We split the dataset randomly into 80% for training and the rest 20% for the testing set. Few samples of the dataset are shown in Fig 8.

### 4.2 Experimental setup and evaluation

To assess the performance of our method, we used accuracy, precision, sensitivity, specificity, and F1-score metrics. Detailed information about the experimental setup and training protocol is given in Table 5.

**Table 5:** Details of experimental setup and training protocols.

Sr. No	Name	Value
1	CPU of Computer system	Intel (R) Core (TM) i5-5200U
2	RAM	8GB
3	HDD	500GB
4	Implementation tool	MATLAB R2020a
5	Window	Window 10, 64 bit
6	Training set	80% data
7	Testing set	20% data

#### 4.2.1 Performance evaluation on brain tumor detection

The goal of this experiment is to assess how well our framework works in spotting brain tumors. For this experiment, we used 3000 MRI scans from the openly available Kaggle BTD-MRI dataset [65], of which 2400 MRI scans (1200 healthy, 1200 tumorous) were used for training and the remaining 600 scans (300 healthy, 300 tumorous) for testing. It took 1533 minutes and 47 seconds to train our proposed model to find brain tumors. Our technique attained a detection accuracy of 99.83%, precision of 100%, F-measure of 99.83%, specificity of 100%, and recall of 99.66%, which proves the effectiveness of our proposed approach for brain tumor detection.

To further verify the robustness and assess the performance of our framework for brain tumor detection on a small-sized dataset, we validated our framework on another widely available Kaggle dataset, i.e., the BMI-BTD dataset [66]. For this experiment, we used all 253 MRI scans where 202 (124 tumorous and 78 normal) were used for training, while the remaining 51 (31 tumorous and 20 normal) were utilized for testing. Our framework was trained in 78 minutes and 4 seconds for the tumor detection task. The results of this experiment are shown in Table 6. Despite the availability of limited data in this dataset, our method achieved an accuracy of 96.08%, precision of 95%, F-measure of 99.83%, specificity of 96.77%, and recall of 95%, which proves the robustness of our approach for brain tumor detection even on a small sized dataset.

**Table 6:** Performance evaluation on brain tumor detection.

Dataset	Accuracy (%)	Precision (%)	Recall (%)	Specificity (%)	F1-score (%)
BTD-MRI	99.83	100	99.66	100	99.83
BMI-BTD	96.08	95	95	96.77	95

Because of the lightweight nature of our model due to employing depthwise separable Conv, it can be easily deployed on mobile or other low-power portable devices for identification and categorization of the tumor in real-time. Our model employs 3×3 depthwise separable convolutions, which need 8 to 9 times less processing time than the traditional convolutions at the cost of a slight decrease in accuracy. Also, the use of BN after convolutional layers speeds up the learning. Furthermore, the images of both datasets are different in terms of formats and resolution. Also, the MRI scans of both datasets are varied in terms of the presence of tumors having variations in angles, intensities, sizes, shapes, and locations. So, we assert that our framework can be effectively utilized for brain tumor detection under these diverse conditions efficiently.

The reason we achieved good performance is that our proposed framework uses both LRelu and ReLu activation functions. ReLU is more computationally efficient because it doesn’t need to perform complicated exponential computations; it merely needs to choose the maximum (0, x). Additionally, we avoided the dying ReLU issue by employing the LReLU AF. The DL model will stop growing when the ReLU problem reaches its end. To solve this problem, we implemented our TumorDetNet technique using a leaky ReLU. The LReLU activation approach allows for a modest (non-zero) gradient while the neuron is not in use. As a consequence, it continues to learn rather than coming to a halt or nearing a dead end. Hence using both AF improve the feature extraction ability of our model, thus makes our model perform more accurately for tumor detection.

#### 4.2.2 Performance evaluation of binary classification of tumor

The purpose of this experiment is to verify the proposed approach’s classification performance for distinguishing benign from malignant tumors. For this experiment, we used 700 MRI scans from the TCD dataset [67], of which 560 were used for training (280 MRI images for each class) and 140 for testing (70 MRI images for each class). It took 316.33 minutes to train our model to distinguish between malignant and benign tumors. Despite the dataset’s sparse data, our system achieved the maximum precision, accuracy, recall, specificity, and F1-score of 100%, demonstrating how well it can distinguish between benign and malignant tumors. To further verify the robustness of our framework for separating benign and malignant brain tumors, we tested our framework on another standard Kaggle dataset BTTypes [68]. The dataset comprises two collections, i.e., benign and malignant, each having 1200 images. For this experiment, we used all the 2400 images (1200 benign and 1200 malignant) in this dataset. Among these MRI images, 1920 images (960 MRI of each class) were used for the training, whereas, the rest of 480 MRI scans (240 MRI of each class) were used for testing. The training process took 1290 minutes and 54 seconds. The results of this experiment are shown in Table 7. Our proposed framework achieved the maximum precision, accuracy, F1-score, and recall of 100% which validates the effectiveness of our approach for brain tumor classification into malignant and benign. Our model achieved the best performance on both datasets due to the ability to extract more important, discriminative, and descriptive deep features for the classification. All convolutional layers of our proposed model use 3×3 and 1×1 kernels with a small stride with the purpose to capture even the smallest texture details (alteration in size, shape, and intensity of brain tumors) of tumors in brain MRI scans.

**Table 7:** Performance evaluation on brain tumor classification into benign and malignant.

Dataset	Accuracy (%)	Precision (%)	Recall (%)	Specificity (%)	F1-score (%)
TCD	100	100	100	100	100
BTTypes	100	100	100	100	100

#### 4.2.3 Performance evaluation of multi-class classification of tumor

To test the multi-class classification ability, we conducted an experiment to evaluate the performance of our TumorDetNet architecture for tumor classification into glioma, pituitary, and meningioma. For this experiment, we used 2764 images (937 meningioma MRI scans, 901 Pituitary MRI scans, and 926 glioma MRI scans) of the standard Kaggle BTC (MRI) dataset [69], where 2212 images (750 meningiomas, 721 Pituitary, and 741 gliomas) were utilized for training and remaining 552 MRI scans (180 Pituitary, 185 gliomas, and 187 meningiomas) for testing. Our model’s training took 1323 minutes and 12 seconds. Our method achieved a precision of 99%, an accuracy of 99.27%, recall of 98.66%, specificity of 100%, and F-measure of 98.82% which proves the usefulness of our approach for brain tumor classification in glioma, pituitary, and meningioma.

To further validate the reliability of our TumorDetNet framework for multi-class classification, we validated our model on another standard CE-MRI figshare dataset [70]. For this experiment, we used all 3064 2D MRI scans of the datasets (i.e., 1426 scans of glioma, 930 scans of the pituitary, and 708 scans of meningioma tumors). Among them, 2451 scans (1141 glioma, 744 pituitary, and 566 meningiomas) were used for the training, and the rest of 613 scans (186 pituitary, 142 meningiomas, and 285 gliomas) were used for testing. The training process took 1517 minutes and 20 seconds. The results are shown in Table 8. The proposed approach achieved a specificity of 96.73%, precision of 98%, recall of 97%, accuracy of 98.47%, and F-measure of 97.49%.

**Table 8:** Performance evaluation on brain tumor classification into glioma, pituitary, and meningioma.

Dataset	Accuracy (%)	Precision (%)	Recall (%)	Specificity (%)	F1-score (%)
BTC (MRI)	99.27	99	98.66	100	98.82
CE-MRI figshare	98.47	98	97	96.73	97.49

The reason for such remarkable performance is that our proposed framework uses the BN technique in the feature map which provides regularization, normalizes the inputs to a layer for each mini-batch, and lowers the generalization error. Moreover, the dropout layer employed in the classification unit of the TumorDetNet framework offers regularization (a generalization of results for new data) by destroying a portion of the outputs from the preceding layer in order to reduce overfitting and enhance generalization.

#### 4.2.4 Brain tumor detection comparison with state-of-the-art DL models

This experiment aims to compare several state-of-the-art (SOTA) DL-based frameworks with the proposed framework for brain tumor identification to assess its effectiveness. In order to do this, we contrasted the detection efficacy of our proposed model to benchmarks developed by ResNet18 [71], DenseNet201 [72], MobileNetv2 [58], and DarkNet53 [73]. Each of these modern DL models was trained using millions of photos from the ImageNet dataset in a TL setup. The last layer of the pre-trained versions of all models was fine-tuned to categorize the images into two classes, i.e., tumorous and healthy. Network input image sizes vary depending on the model, for example, darknet53’s input image size is 227 by 227 whereas resnet18’s is 224-by-224. To comply with each comparative DL model’s requirement, we adjusted the input image size. For this experiment, we used the freely available Kaggle BTD-MRI database [65], which included 600 MR scans (300 MR scans of each healthy and tumor class) and 2400 MR scans (1200 MR scans of each healthy and tumor class). The results are given in Table 9. From these results, it is clear that our TumorDetNet framework attained the highest results for almost all performance metrics as compared to all four contemporary models by achieving a precision of 100%, accuracy of 99.83%, specificity of 100%, recall of 99.66%, and F-score of 99.83% for brain tumor detection. The accuracy of the Mobilenetv2 model was 99%, which was the second-best result, whereas, DenseNet201 attained the lowest accuracy of 95% among all frameworks. It is to be noted that the detection accuracy of all comparative frameworks was above 95%. It is significant to note that the precision and specificity of our approach are 100%. These results demonstrate the efficacy of our model for detecting brain tumors when compared to other models.

**Table 9:** Brain tumor detection comparison with SOTA models.

Model	Accuracy (%)	Precision (%)	Recall (%)	Specificity (%)	F1-score (%)
Resnet18	98.17	97.00	99.32	95.65	98.15
Denenet201	95.00	98.00	92.45	97.87	95.15
Mobilenetv2	99.00	99.33	98.68	99.33	99.00
Darknet53	96.17	97.33	95.11	97.27	96.21
**Proposed TumorDetNet**	99.83	100	99.66	100	99.83

#### 4.2.5 Brain tumor binary classification comparison with SOTA DL models

The key purpose of this experiment is to evaluate the worth of our TumorDetNet framework for brain tumor classification into malignant and benign over the different SOTA DL-based frameworks. To do this, we contrasted the performance of our model with the ResNet18 [71], MobileNetv2 [58], Darknet19 [74], and DarkNet53 [74] on the TCD dataset. In a TL configuration, each of these contemporary DL models was trained on the ImageNet dataset. The last layer of the pre-trained versions of all networks was fine-tuned to categorize the images into two categories, i.e., benign and malignant. Again, because of differences in the input image sizes of different frameworks, we adjusted the input MRI scan size to satisfy the requirements of each DL framework. For this experiment, we used 700 MRI scans from the freely available TCD dataset [67], of which 560 were used for training (280 for each class) and the remaining 140 were used for testing (70 for each class). Table 10 presents the findings. From these data, it is evident that our model, when compared to all other modern models, produced the greatest outcomes by correctly classifying brain tumors as malignant or benign with 100% precision, accuracy, specificity, F-score, and recall. In contrast to resnet18 and mobilenetv2, which both achieved the lowest accuracy of 96.43% across all networks, the Darknet53 framework achieved the second-best accuracy of 99.29%. This comparative analysis demonstrates that our approach outperforms other methods for classifying malignant and benign tumors.

**Table 10:** Comparative analysis of brain tumor classification into benign and malignant with SOTA models.

Model	Accuracy (%)	Precision (%)	Recall (%)	Specificity (%)	F1-score (%)
Resnet18	96.43	100	93.33	100	96.55
Mobilenetv2	96.43	1.00	93.33	100	96.55
Darknet53	99.29	98.57	100	98.59	99.29
Darknet19	97.14	100	94.59	100	97.22
Proposed TumorDetNet	100	100	100	100	100

#### 4.2.6 Brain tumor multi-class classification comparison with SOTA DL models

The aim of this experiment is to evaluate the usefulness of our framework for multiclass classification (i.e., meningioma, pituitary, and glioma, etc.) of brain tumors over these contemporary DL-based models i.e., ResNet18 [71], Resnet50 [71], Denenet201 [65], Darknet19 [74] and MobileNetv2 [58] on the BTC dataset. All of these contemporary DL models were used in a TL setup as discussed in previous experiments. The last layer of the pre-trained versions of all networks was fine-tuned to categorize the MRI scans into three classes, i.e., glioma, pituitary, and meningioma. As with prior experiments, we altered the input image size to fit the requirements of each DL framework. For this experiment, we used 2764 images (937 meningiomas, 901 Pituitary, and 926 gliomas) of the BTC (MRI) dataset [69], where 2212 images (750 meningiomas, 721 Pituitary, and 741 gliomas) were utilized for training and outstanding 552 MRI scans (180 Pituitary, 185 gliomas, and 187 meningiomas) for testing. The findings are given in Table 11. From these results, it is confirmed that our approach achieved the best results for all performance metrics as compared to all contemporary models by achieving a precision of 99%, accuracy of 99.27%, recall of 98.66%, specificity of 100%, and F-measure of 98.82%, which proves the efficacy of our proposed approach for brain tumor categorization into pituitary, glioma, and meningioma. The TL of resnet50 attained the second-best accuracy of 98.73%, whereas, darknet19 attained the lowermost accuracy of 70.47% among all frameworks. It is important to mention that our framework obtained the optimal 100% specificity. These results demonstrate that our framework outperforms other comparative models for the multiclass classification of tumors.

**Table 11:** Comparative analysis of brain tumor classification into meningioma, pituitary, and glioma with SOTA models.

Model	Accuracy (%)	Precision (%)	Recall (%)	Specificity (%)	F1-score (%)
Resnet18	93.24	89.66	92.33	90.34	89.33
Resnet50	98.73	98.66	98.66	98.91	98.66
Mobilenetv2	97.34	96	96	96.21	96
Densenet201	89.12	83.66	84	90.38	83.66
Darknet19	70.47	71	69.333	75.53	70
**Proposed TumorDetNet**	99.27	99	98.66	100	98.82

### 4.2.7 Brain tumor detection and classification comparison with existing approaches

We designed a multi-stage experiment to give a thorough comparison of our technique with existing SOTA brain tumour identification and classification approaches in order to demonstrate the superiority of our framework over existing methods. To do this, we compared our technique to the most current strategies [74–79] and presented the findings in Table 12. First, we made a comparison between our model and the most recent techniques for finding brain tumours [74–76]. The findings are presented in Table 12 (rows 1–3). In the second stage, we compared our model with the contemporary brain tumor classification (meningioma, pituitary, and glioma) methods [77–79] and the results are shown in Table 12 (row 5 to row 7). Table 12 shows a comparative analysis based on the classification performance. The comparison demonstrates that the proposed performance outperforms all the SOTA approaches. We included only accuracy as a performance metric in this experiment as used by the comparative methods. Furthermore, to prove the efficacy, reliability, and superiority of our method, we compared our approach with SOTA approaches for those datasets on which we obtained lower performance. The proposed framework performed the best and reached an accuracy of 96.08% in the first example, [75] earned the second-highest accuracy of 96.05%, and [74] achieved the lowest accuracy of 89.8% for brain tumour identification. In the second instance, our framework produced the best outcomes with an accuracy of 98.47%, [79] produced the second-best results with an accuracy of 98.21%, and [78] produced the worst results with an accuracy of 96.6% for the categorization of brain tumours into meningioma, glioma, and pituitary. The results of this experiment evidently prove that our model provides superior brain tumor detection and classification performance over the contemporary methods. It is important to note that, unlike the comparative methods that either performs the detection or classification of brain tumors, we proposed a unified method capable of both the detection, and binary and multi-class classification of brain tumors. Compared to previous works, the proposed DL framework attained the highest detection and classification performance on six diverse and standard datasets, including a small-sized dataset and unbalanced data samples in two datasets, available at Kaggle.

**Table 12:** Comparative analysis of the proposed and SOTA methods.

SN	Methods	Method	Dataset	Year	Accuracy (%)
Brain tumor detection					
1	Kiraz et al. [74]	weighted KNN	BMI-BTD	2021	89.8
2	Togacar [75]	BrainMRNet	BMI-BTD	2019	96.05
3	Saxena et al. [76]	ResNet-50	BMI-BTD	2019	95.0
4	**Proposed study**	TumorDetNet	BMI-BTD		96.08
**Brain tumor classification into meningioma, pituitary & glioma**					
5	Kibriya et al. [77]	13-layer CNN	CE-MRI	2022	97.2
6	Demir et al. [78]	Residual-CNN	CE-MRI	2022	96.6
7	Kesav et al. [79]	RCNN with Two Channel CNN	CE-MRI	2021	98.21
8	**Proposed study**	TumorDetNet	CE-MRI		98.47

#### 4.2.8 Cross dataset validation

The key aim of this experiment is to analyze the generalization power of our approach for brain tumor classification into malignant and benign. For this purpose, we designed a cross-dataset evaluation for the following scenarios: (a) trained our model on the 2400 images (1200 benign and 1200 malignant) of BTTypes dataset and tested it over the 700 images (350 benign and 350 malignant) of the TCD dataset, (b) trained our model on the 700 images (350 benign and 350 malignant) of the standard TCD dataset and tested it over the 2400 images (1200 benign and 1200 malignant) of BTTypes dataset. Both datasets consist of two classes i.e., malignant and benign. The images of both datasets are diverse as they contain tumors of different shapes, angles, sizes, intensities, and placements in the brain. Images of both datasets have different formats, bit depth, and resolution. The images of BTTypes dataset are in PNG format, with a bit depth of 32, and a resolution of 224×224 whereas the MRI scans of the TCD is in JPG format, with a bit depth of 32, and a resolution of 203×170. In spite of training on one dataset and testing on unseen samples of a completely different dataset, our approach achieved excellent precision, accuracy, recall, F1-score, and specificity of 99% for both scenarios showing the excellent generalizability of our model for the brain tumor classification into malignant and benign tumors.

## 5. Conclusion

This work has presented a unified TumorDetNet DL model for the automated detection and classification of brain tumors. The presented DL-based model reliably detects brain tumor and classify the brain tumors into two i.e., malignant and benign, and three types i.e., meningioma, pituitary, and meningioma. The robustness of our methodology has also been verified using six publically accessible datasets. The proposed framework’s superiority over the existing methods has been confirmed by its accuracy of 99.83% for brain tumor detection, optimal accuracy of 100% for classifying brain tumors into malignant and benign, and accuracy of 99.27% for classifying brain tumors into pituitary, glioma, and meningioma. Experimental findings demonstrate that our framework outperforms contemporary brain tumor detection and classification techniques. In comparison to existing methods, our suggested method had the greatest accuracy for brain tumor identification and classification while requiring less pre-processing. Additionally, cross-corpora examination of our technique demonstrates its applicability to the classification of brain tumors in particular. Despite the promising findings of the proposed technique, our work has potential for improvement in a few aspects. When using other imaging modalities, such as computer tomography (CT) scans, the proposed TumorDetNet technique does not reveal how effectively the system can identify brain tumors. The proposed model is complex and requires more time to detect and classify brain tumors. Moreover, the performance of our method has not been verified in genuine clinical investigations, despite the proposed technique doing very well on several publicly accessible datasets. We want to further minimize our model’s computing time, memory space, and system complexity in the future. This work can be further extended to identify and categorize more diseases as well as other complex types of tumors.
